# Periodic Characteristics of Hepatitis Virus Infections From 2013 to 2020 and Their Association With Meteorological Factors in Guangdong, China: Surveillance Study

**DOI:** 10.2196/45199

**Published:** 2023-06-15

**Authors:** Xixi Zhao, Meijia Li, Naem Haihambo, Xinni Wang, Bin Wang, Meirong Sun, Mingrou Guo, Chuanliang Han

**Affiliations:** 1 The National Clinical Research Center for Mental Disorders and Beijing Key Laboratory of Mental Disorders Beijing Anding Hospital Capital Medical University Beijing China; 2 Advanced Innovation Center for Human Brain Protection Capital Medical University Beijing China; 3 Faculty of Psychology and Center for Neuroscience Vrije Universiteit Brussel Brussel Belgium; 4 Faculty of Psychology Beijing Normal University Beijing China; 5 School of Psychology Beijing Sport University Beijing China; 6 The Brain Cognition and Brain Disease Institute Shenzhen Institute of Advanced Technology Chinese Academy of Sciences Shenzhen China; 7 The Guangdong Provincial Key Laboratory of Brain Connectome and Behavior Shenzhen China; 8 Chinese Academy of Sciences Key Laboratory of Brain Connectome and Manipulation Shenzhen China; 9 Shenzhen-Hong Kong Institute of Brain Science-Shenzhen Fundamental Research Institutions Shenzhen China

**Keywords:** hepatitis virus, meteorological factors, epidemics, recrudescence, public health

## Abstract

**Background:**

In the past few decades, liver disease has gradually become one of the major causes of death and illness worldwide. Hepatitis is one of the most common liver diseases in China. There have been intermittent and epidemic outbreaks of hepatitis worldwide, with a tendency toward cyclical recurrences. This periodicity poses challenges to epidemic prevention and control.

**Objective:**

In this study, we aimed to investigate the relationship between the periodic characteristics of the hepatitis epidemic and local meteorological elements in Guangdong, China, which is a representative province with the largest population and gross domestic product in China.

**Methods:**

Time series data sets from January 2013 to December 2020 for 4 notifiable infectious diseases caused by hepatitis viruses (ie, hepatitis A, B, C, and E viruses) and monthly data of meteorological elements (ie, temperature, precipitation, and humidity) were used in this study. Power spectrum analysis was conducted on time series data, and correlation and regression analyses were performed to assess the relationship between the epidemics and meteorological elements.

**Results:**

The 4 hepatitis epidemics showed clear periodic phenomena in the 8-year data set in connection with meteorological elements. Based on the correlation analysis, temperature demonstrated the strongest correlation with hepatitis A, B, and C epidemics, while humidity was most significantly associated with the hepatitis E epidemic. Regression analysis revealed a positive and significant coefficient between temperature and hepatitis A, B, and C epidemics in Guangdong, while humidity had a strong and significant association with the hepatitis E epidemic, and its relationship with temperature was relatively weak.

**Conclusions:**

These findings provide a better understanding of the mechanisms underlying different hepatitis epidemics and their connection to meteorological factors. This understanding can help guide local governments in predicting and preparing for future epidemics based on weather patterns and potentially aid in the development of effective prevention measures and policies.

## Introduction

Hepatitis, which describes an inflammation of the liver, is one of the most common diseases in the world. Hepatitis can be caused by a variety of factors. However, viruses are the most prevalent cause. Hepatitis A [1-4], B [5-9], C [10-14], and E [1,15-18] are the 4 most common types of hepatitis in China, and they are caused by the hepatitis A [19,20], B [21], C [22,23], and E viruses (HAV, HBV, HCV, and HEV), respectively. The hepatitis viruses can survive for a long time in a variety of harsh environments and have a strong resistance to external factors [24]. Hepatitis A and E are transmitted through the digestive tract via fecal-oral transmission and contaminated food or water [25,26]. Hepatitis B and C are spread through the blood or bodily fluids of an infected person, mother-to-child transmission, and sexual transmission [8,27-29]. In 2020, the number of confirmed cases of hepatitis A, B, C, and E in Guangdong Province was 1594, 18,4524, 28,068, and 2070, respectively. This represents an increase of 1.10 times, 1.15 times, 1.23 times, and a decrease of 0.75 times, respectively, compared to statistics from 2013. Hepatitis A, B, and C showed an upward trend, whereas hepatitis E showed a downward trend.

Viruses can be transmitted in 2 ways: horizontal and vertical transmission. Horizontal transmission involves the transmission of a virus from one person to another within the same generation, while vertical transmission refers to the transmission of viruses from mother to child. Hepatitis infections often show an age-dependent property, with more severe symptoms observed in adults [19]. People exposed to HBV through horizontal transmission often develop self-limited acute infections, while people who contract the virus from their mothers through vertical transmission tend to become chronic HBV carriers [21]. This difference in infection severity may be due to the difference in immune response between adults and young children [21]. The infection of hepatitis also showed genotype-dependent properties. For example, in HEV, there are 4 genotypes (HEV 1-4) [30]. The prevalence of HEV1 and HEV2 in developing regions have led to large-scale outbreaks. If infection occurs during pregnancy, it leads to severe hepatitis. In contrast, HEV3 and HEV4 are mainly observed in developed regions and are common in both humans and animals, with pigs as the main host [15].

Recrudescence at a fixed frequency is a common feature of infectious diseases throughout the world [31-34]. Previous research has shown that hepatitis epidemics in China exhibit oscillatory properties at a national level [35,36]. Natural forces (eg, temperature [37] and natural disasters [38]) might drive this oscillatory infection. Several examples have shown that epidemics, such as measles [39], pertussis [40-44], influenza [45], and rabies [46-48], can have oscillations. However, whether the oscillatory phenomena of hepatitis epidemics were influenced by environmental factors remains unclear.

In light of the above, in this study, we aimed to explore the oscillatory properties of the hepatitis epidemic in Guangdong (20°13'-25°31' N and 109°39'-117°19' E) as well as the potential natural contributors to the oscillatory outbreaks. We obtained data for the period from January 2013 to December 2020 on 4 notifiable infectious diseases caused by hepatitis viruses (including HAV, HBV, HCV, and HEV) in Guangdong and the meteorological elements (eg, temperature, precipitation, and humidity) in the same time frame. Power spectrum analysis was conducted on these data to capture the oscillatory strength of the outbreaks. We then explored the relationship between meteorological elements and oscillatory properties based on the regression and correlation analyses.

## Methods

### Data and Sources

Time series data on available monthly reported and confirmed cases of 4 hepatitis diseases (A, B, C, and E) were obtained for Guangdong province in China’s mainland, from January 2013 to December 2020, from the Health Commission of Guangdong. The data set is available to the public around the world and is reported monthly. Monthly reported data of meteorological elements (eg, temperature, precipitation, and humidity) of Guangdong province from January 2013 to December 2020 were obtained from the China Statistical Yearbook 2014-2021. The meteorological factors included in this study are continuous values and vary with time, which would directly reflect the actual natural conditions each month. This data set is also available to the public around the world and is reported annually.

### Ethical Considerations

For this study, the data we used are open to the public. Our study did not involve any interventions in human participants. This study was approved by the ethics committee of Beijing Sport University, China (2022142H).

### Power Spectrum Analysis

We used spectrum analysis to quantify fluctuations and the recurrence of epidemics. Similar methods have been used in classic and modern studies in the field of public health [35,36,49], medicine [50-53], and biological research [54,55]. The power spectral density for each infectious disease was computed via the multitaper method using the Chronux toolbox [56], an open-source data analysis toolbox [57]. The definition of the peak in the power spectrum is the ratio of the power in a specific frequency (eg, once or twice a year) and the power in the surrounding frequencies (0.75-1.25 and 1.75-2.25). In this study, a ratio larger than 3 was considered an obvious oscillatory peak.

### Tuning Curves for Monthly Infected Cases

The tuning curve of monthly infected cases depicts the basic character of disease outbreaks, providing a direct view of the situation each month based on the historical data. We took the monthly average number of infected cases of each hepatitis epidemic and computed them into a tuning curve (equation 1). Each type of hepatitis epidemic in this study has a tuning curve, and the periodic pattern within a year would be obvious based on it.



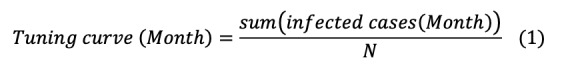



In this equation, N represents the number of the year.

### Tuning Curves for Meteorological Factors

The tuning curve of meteorological factors (eg, temperature, precipitation, and humidity) depicts the basic character of natural conditions, providing a direct view of the situation each month based on historical data. We computed the monthly average of meteorological factors into a tuning curve (equation 2). Each meteorological factor in this study has a tuning curve, and the periodic pattern within a year is obvious based on it.



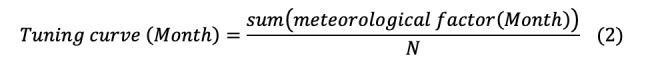



In this equation, N represents the number of the year.

### Regression Analysis

The regression model was shown as equation 3.



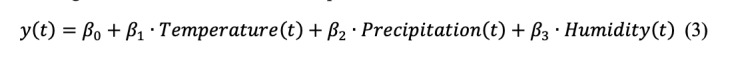



In this equation, *β* represents the regression coefficients of the model, and *y(t)* represents the number of cases with hepatitis infection. The regression analysis was conducted using the regress function in MATLAB (2020a) [58].

### Correlation Analysis

We used the Pearson correlation to measure the relationship between infected cases and meteorological elements. The correlation analysis was conducted using the corr function in MATLAB (2020a).

## Results

### Periodic Phenomena of Hepatitis Epidemics in Guangdong, China

**Figure 1:**
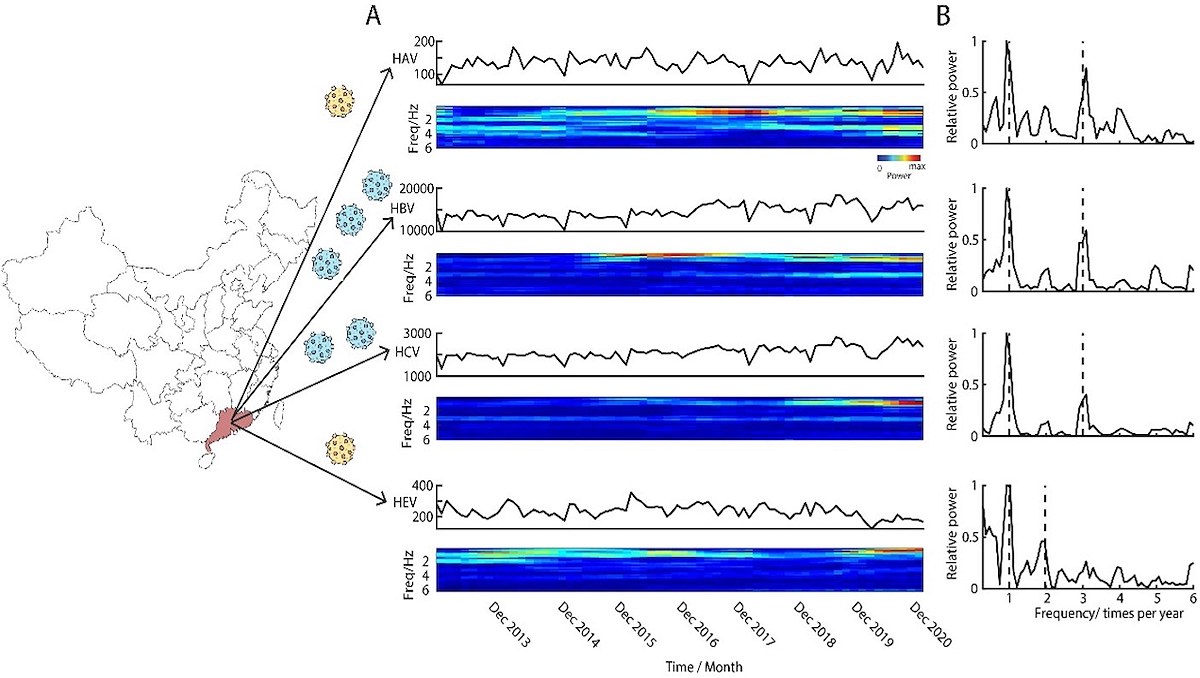
Periodic phenomena of hepatitis epidemics with power spectrum. Left map shows the geological location of Guangdong. (A) shows the monthly incidences of hepatitis A, B, C, and E viruses (HAV, HBV, HCV, and HEV) and its spectrogram from January 2013 to December 2020 in Guangdong. (B) The power spectrum of time series data shown in panel A. The y-axis is the relative power, which is defined as power(f)/max(power(f)).

This study analyzed the monthly data of confirmed cases of 4 hepatitis diseases in Guangdong from January 2013 to December 2020 (Figure 1). Time series data of the 4 hepatitis epidemics (A, B, C, and E) in Guangdong is shown in Figure 1A. It is clear that all hepatitis epidemics in Guangdong have obvious oscillatory patterns based on their power spectrum (Figure 1B), which shows different peaks (eg, peaking once, twice, or 3 times a year). However, for the oscillatory patterns, the hepatitis E epidemic (the fourth row in Figure 1B) differs from the other 3 hepatitis epidemics (first 3 rows in Figure 1B). Hepatitis A, B, and C epidemics show 2 main peaks (occurring once and 3 times per year) in the power spectrums, whereas the hepatitis E epidemic, in addition to the cycle of outbreaks once a year, will have another cycle of outbreaks twice a year.

### Periodic Phenomena of Meteorological Elements in Guangdong, China

**Figure 2:**
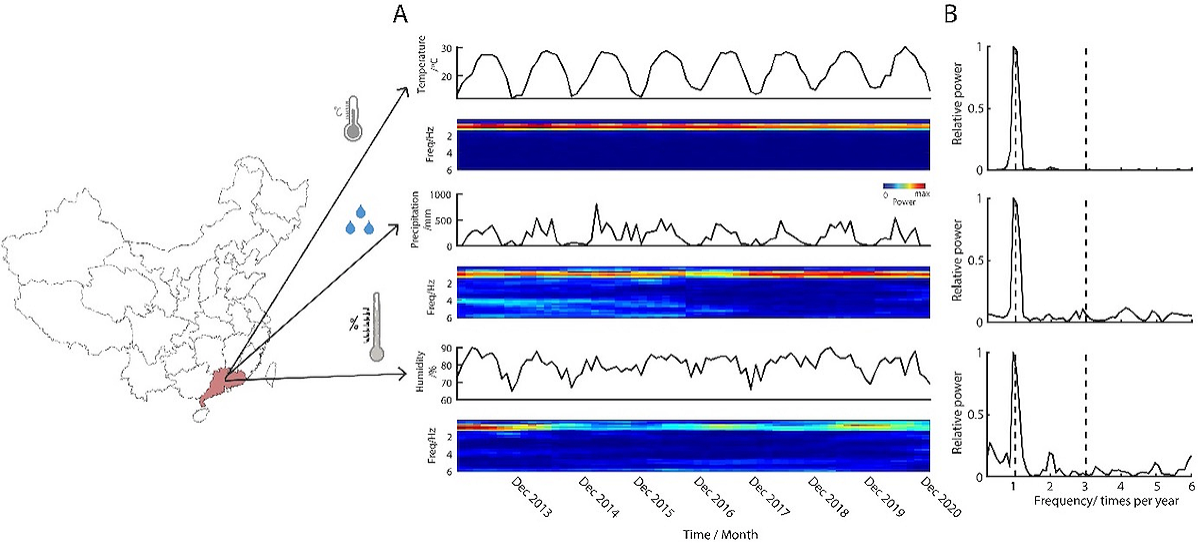
Meteorological elements of hepatitis epidemics with power spectrum. Left map shows the geological location of Guangdong. (A) The monthly time series meteorological elements (temperature, precipitation, and humidity) and its spectrogram from January 2013 to December 2020 in Guangdong. (B) The power spectrum of time series data shown in panel A. The y-axis is the relative power, which is defined as power(f)/max(power(f)).

Meteorological elements might be a potential contributor to the periodic phenomenon of the epidemic; therefore, we also conducted the power spectrum analysis of the time series data of temperature, precipitation, and humidity in Guangdong province (Figure 2A and Figure 2B). All 3 meteorological elements in Guangdong exhibit evident oscillatory patterns throughout time, indicating that the epidemics and meteorological elements may be correlated.

### Relationship Between the Periodic Characteristics of Hepatitis Infection and Meteorological Elements in Guangdong, China

**Figure 3:**
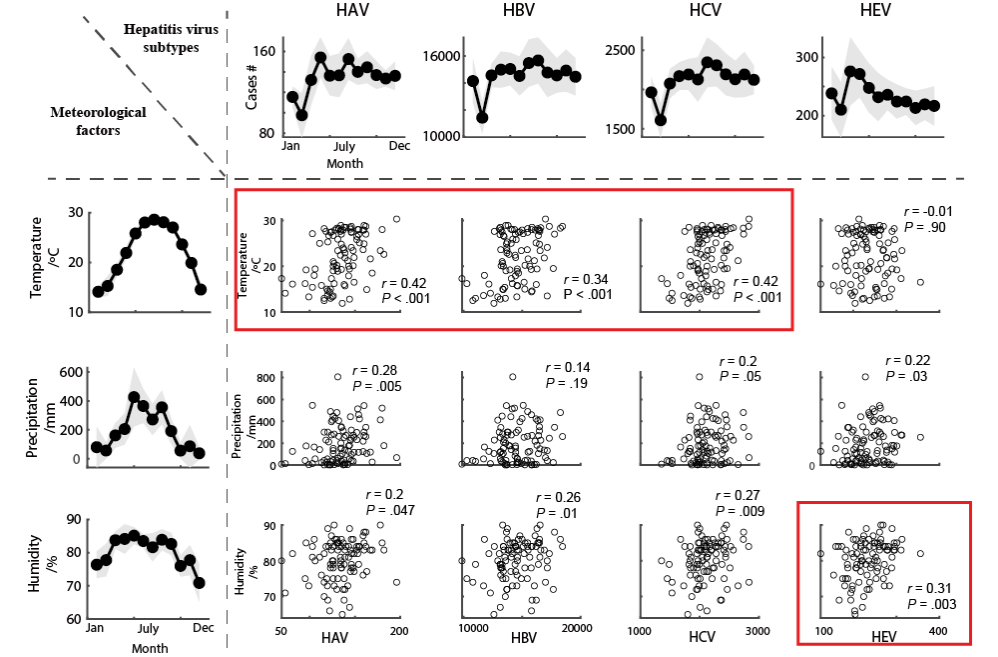
Relationship between infected cases of hepatitis epidemic and meteorological elements. Top row: the tunning curves for the number of infected cases of 4 hepatitis epidemics over a year, with the grey area denoting the standard error of mean. Left column: the tunning curves for an index of natural factors for each month of each year. Rows 2-4 show scatter plots between the number of cases of each hepatitis type and temperature, precipitation, and humidity, respectively. Each column represents a different type of hepatitis. The red square in each plot indicates conditions that showed significant results in both correlation and regression analyses. HAV: hepatitis A virus; HBV: hepatitis B virus; HCV: hepatitis C virus; HEV: hepatitis E virus.

From the observation of the average infected cases of the hepatitis epidemic (first row in Figure 3) and the average meteorological elements (first column in Figure 3), hepatitis infections may be influenced by several natural factors. We then investigated the relationship between hepatitis epidemics and meteorological elements in Guangdong in detail based on multiple analysis approaches.

From the correlation analysis, we discovered a substantial positive association between temperature and the incidence of HAV (*P*<.001), HBV (*P*<.001), and HCV (*P*<.001), showing that the warmer the temperature, the more infected patients there are. Precipitation was positively correlated with HAV (*P*=.005) and HEV (*P*=.03), indicating that the more rainfall there is, the higher the incidence of HAV and HEV. Humidity was positively correlated with HAV (*P*=.047), HBV (*P*=.01), HCV (*P*=.009), and HEV (*P*=.003), showing that the more humid the environment is, the greater the prevalence of hepatitis A, B, C, and E (Figure 3). No additional significant findings were identified. The correlation analysis revealed that temperature had the most significant correlation with HAV, HBV, and HCV, while humidity had the most significant correlation with HEV (depicted by the red square in Figure 3). Since humidity is often affected by precipitation, to further validate their relationship and exclude the influence of other factors (eg, precipitation), we built 2 regression models to investigate how meteorological factors predict the number of hepatitis infections, with and without precipitation as a variable.

From the regression analysis between the number of hepatitis infections and meteorological factors (Table 1), we first checked collinearity by variance inflation factor.

All the variance inflation factors are in the range from 1.5 to 2 (ie, 1.64, 1.93, and 1.78). The correlation coefficients among the 3 meteorological factors (ie, temperature, precipitation, and humidity) were lower than 0.8 (temperature and precipitation: 0.59; temperature and humidity: 0.54; and precipitation and humidity: 0.63, respectively), which would not be affected by collinearity. In the first regression model, using 3 meteorological factors to predict the number of hepatitis infections, the coefficients of temperature in the prediction of hepatitis A, B, and C were significantly positive (hepatitis A: *F*=6.995; *P*<.001, hepatitis B: *F*=5.428; *P*=.001, and hepatitis C: *F*=8.66; *P*<.001) but not significant for the coefficients of precipitation and humidity, suggesting that temperature can positively predict the incidence of hepatitis A, B, and C and is the main contributor to these 3 epidemics. In terms of the hepatitis E epidemic (*F*=4.74; *P*=.004), we found a significant positive coefficient for humidity (*P*=.004) and a weak but significant negative coefficient for temperature (*P*=.03), indicating that a more humid and less warm environment can predict higher incidences of hepatitis E. The second regression model that excluded precipitation as a predictor showed a result that was consistent with the model with 3 predictors, except that the significance level was more robust for humidity to predict HEV infections (Table 2).

In sum, we found that warmer temperatures can predict a higher prevalence of hepatitis A, B, and C in Guangdong. Meanwhile, higher humidity and lower temperature can both predict a higher prevalence of hepatitis E.

**Table 1:** Statistics for the regression analysis (temperature, precipitation, and humidity).

Regression anlysis	Hepatitis A virus		Hepatitis B virus			Hepatitis C virus			Hepatitis E virus	
	Coefficient	*t* value		Coefficient	*t* value		Coefficient	*t* value	Coefficient	*t* value
β_0_	106.94	—^a^		10320.51	—		1425.52	—	49.96	—
β_1(temperature)_	1.809	3.71^b^		127.78	3.29^c^		27.59	4.41^b^	–2.05	2.2^d^
β_2__(precipitation)_	0.001	0.04		–1.04	0.77		–0.22	1.02	0.025	0.76
β_3(humidity)_	–0.155	0.31		20.01	0.51		1.57	0.24	2.80	2.93^c^

^a^Not applicable.

^b^*P*<.001.

^c^*P*<.01 (β_1(temperature)_ for HBV: *P*=.001; β_3(humidity)_ for HEV: *P*=.004).

^d^*P*<.05 (*P*=.03)

**Table 2:** Statistics for the regression analysis (temperature and humidity).

Regression anlysis	Hepatitis A virus		Hepatitis B virus			Hepatitis C virus			Hepatitis E virus	
	Coefficient	*t* value	Coefficient	*t* value	Coefficient		*t* value	Coefficient		*t* value
β_0_	106.23	—^a^	11489.05	—	1675.11		—	21.88		—
β_1(temperature)_	1.816	4.06^b^	116.365	3.25^c^	25.15		4.34^b^	–1.78		–2.03^d^
β_2(humidity)_	–0.146	–0.34	6.113	0.18	–1.39		–0.25	3.13		3.70^b^

^a^Not applicable.

^b^*P*<.001.

^c^*P*<.01 (*P*=.001).

^d^*P*<.05 (*P*=.04).

## Discussion

### Principal Findings

In this study, we found that hepatitis epidemics (A, B, C, and E) have different oscillatory properties, and hepatitis A, B, and C in Guangdong have a stronger association with temperature, while hepatitis E showed a stronger association with humidity. Different types of hepatitis will have different periodic characteristics and different associations with different natural environments, which is crucial for epidemic prevention and management. Understanding the temporal characteristics of infectious diseases is essential for effective epidemic prevention, as it may inform the development and implementation of appropriate policies and strategies.

### Comparison With Prior Work

To our knowledge, this is the first study to investigate the periodic characteristics of the hepatitis epidemics and their relationship with natural factors, which fills a gap in this field. Previous works have mainly focused on descriptive statistics, detailing the number of infections without describing the precise time characteristics [1,3,18], such as oscillations. Some studies showed that hepatitis epidemics have strong oscillations at a national level [36], but they have not been refined to a more detailed geographical scale (eg, at province levels).

Another issue is the influence of meteorological factors on hepatitis epidemics. HAV and HEV are 2 distinct viruses. Although HAV and HEV share similarities in their fecal-oral transmission route [25,26], our results demonstrate that hepatitis A and E epidemics have different relationships with meteorological factors (hepatitis A epidemic being associated with temperature and hepatitis E epidemic being associated with humidity). This result indicates that even though the 2 viruses share similar transmission modes, the relationship with natural indicators is not necessarily the same, which further indicates that they have unique transmission mechanisms that are not shared, such as host ranges and genotypes.

First, the host range of HAV is limited to humans and several nonhuman primates [59]. There is a tendency for people to engage in more social activities during warmer weather, which increases the likelihood of HAV transmission. In contrast, HEV infects a variety of animal species, including deer, rabbits, and mollusks, but it primarily infects pigs [15,30,60]. Higher humidity is harmful to the health of pigs [61], which will increase contact between pigs and people employed in occupations related to pigs, leading to an increase in HEV infections among the animals and humans who come into contact with an infected animal. Additionally, runoff from outdoor pig farms can contaminate surface water as well as crops that receive this surface water [60]. Higher humidity can increase the risk of HEV contamination of surface water from outdoor pig farms and other sources, and heavy rainfall can lead to the spread of contaminated water to crops and other areas, potentially increasing the incidence of HEV infections. For these reasons, it might be of general value to further investigate water contamination during periods of high rainfall to better understand this phenomenon. Nonetheless, it remains important to implement measures to prevent contamination of water sources and monitor water quality, especially in areas with high levels of outdoor pig farming or other potential sources of contamination.

Second, there are 4 main genotypes of HEV. Genotypes 1 and 2 cause outbreaks or epidemics in humans [62], while genotypes 3 and 4 primarily infect various mammalian species, such as pigs and sheep, but can also infect humans and lead to sporadic cases of hepatitis E. Climate change can affect the environment [63], potentially impacting the quality of water and food sources. For example, the outbreak of HEV genotype 4 infection is more likely to be caused by contaminated tap water rather than contaminated food and contaminated water network on days with heavy rainfall [64]. However, this is not the case for HAV.

Further research is needed to confirm these hypotheses using detailed biological experiments. Previous studies on how temperature affects viral transmission have been equivocal. For example, norovirus prevalence was associated with low water temperatures [65,66], but enterovirus 71 [67] infections began to rise at temperatures above 13°C and declined at temperatures higher than approximately 26°C, exhibiting a V-shaped relationship with the temperature. This phenomenon indicates that transmission patterns are likely influenced by a combination of biological, environmental, and behavioral factors.

### Limitations

One limitation of our study is the limited data. Despite being a representative province, our study only includes a single province in China. In the future, we will further obtain more data and apply similar methods to assess infectious diseases in more regions with varying climates. Even though we have made explorations, several factors remain unclear and cannot be addressed at present. For example, hepatitis A and E have similar propagation principles, but their oscillatory strength is very different in Guangdong Province. In the future, higher-dimensional data will be required to provide more clarity. Another potential limitation is that we did not explore the interaction of the variables and the potential nonlinear relationships in the regression model, which should be considered in other studies in the future, where necessary.

### Conclusions

Our study makes a link between climate change and the recurrence of infectious disease epidemics, enabling us to predict the magnitude of the epidemics based on various weather conditions. It is essential for local meteorological and medical departments to collaborate closely to prepare for the prevention and control of various epidemics. Additionally, the general public should be informed about the impact of different weather conditions on the spread of disease so that they can take appropriate measures to reduce the spread of viruses.

## Abbreviations

HAV: hepatitis A virus
HBV: hepatitis B virus
HCV: hepatitis C virus
HEV: hepatitis E virus

## Data Availability

The data set is available to the public, which can be found on the official website of the Guangdong Provincial Health Commission [68].
